# A lot is done and more will come

**DOI:** 10.1007/s00404-022-06868-8

**Published:** 2022-12-13

**Authors:** Karl Oliver Kagan

**Affiliations:** Department of Women’s Health, University of Tuebingen, Calwerstrasse 7, 72076 Tuebingen, Germany

Dear Reader,

We look back to a great year with many changes.

In January, I took over from Prof. Ortmann as Editor in Chief of Archives of Gynecology and Obstetrics. He gave me the task of developing an already well-run journal.

To this end, it was initially important for us to place the management function on several shoulders. I am therefore very grateful that we were able to recruit Prof. Nicole Sänger, Prof. Bernhard Krämer and Prof. Andreas Hartkopf. They are currently collaborating as Deputy Editors in Chief and covering the subject areas of reproductive medicine, general gynecology, and oncology, while the overall management of the journal and the subject area of prenatal and obstetric medicine is in my hands. All submitted manuscripts are assigned to us for a first review. Only papers with a realistic chance of being accepted are then re-assigned to Associate Editors who manage the peer review process.

We are also very grateful to Dr. Bettina Walter-Thorwart, who supports the journal as Managing Editor, and to Ms Barbara Pedrotti, who is our essential link to Springer.

It would not be possible to manage and develop the journal without these strong pillars.

Of course, the Associate Editors are crucial for a timely evaluation of the manuscripts. Our group includes now more than 25 experts in their field in gynecology and obstetrics. They invite reviewers, evaluate the manuscript based on the reviewers’ assessments and provide recommendations to Nicole, Bernhard, Andreas and me who make the final decision. A special thanks also goes to our Reviewers. They ensure that the peer review process is filled with life and that only high-quality papers are accepted. 698 diverse colleagues have participated in the peer review process in 2022. We are particularly grateful to Prof. Katharina Rall, Prof. Markus Hoopmann, Dr. Susanne Schüler, Dr. Emel Canaz and Dr. Felix Neis, who have done more reviews than anyone else this year. We work hard to reduce the turnaround time and we are on a good way. On average, in 2022, we provided a first decision within 38 days after submission compared to 48 days in the previous year.

In the last 12 months, we have received more than 2,800 manuscripts from all over the world. We would like to thank all the authors who consider publishing their work with us. Unfortunately, we had to reject 82% of the submitted manuscripts. I know how painful and unsatisfying the rejection of a paper can be with its significant burden of research commitment. However, we can only accept a small proportion of the papers that are delivered to us. Still, we would like to encourage everyone to send us their papers; we will take care of them in a timely and professional manner.

IF (2021) reached a value of 2.493. We are indebted to those authors who contributed to the rise of IF with their outstanding papers and to our Editors and Reviewers for their hard work and the restless commitment in selecting the best journal contributions for publication. Take the opportunity to read the most important journal articles, which are available free of charge at https://www.springer.com/journal/404/updates/23115614.

The marked growth of yearly article downloads shows the rising impact of the journal in the research community: + 35% in 2020, + 41% in 2021 and a further 27% increase in usage is expected in 2022. We aim to reach the milestone of 1,000,000 yearly downloads in 2023.

We invite you to stay updated and have a look at what your peers are reading most at https://www.springer.com/journal/404/updates/23115864.

The Journal social media attention is significantly rising over the most recent years: + 20% in 2020, + 31% in 2021 and we expect to double mentions in 2022 compared to the previous year. Since we are aware that social media are playing a more and more active role in academic research, in 2022 we welcomed our Associate Editor, Prof. Florin-Andrei Taran, as Journal Social Media Editor. *Archives of Gynecology and Obstetrics* is now active in Twitter at https://twitter.com/ArchGynObs and a monthly report on more mentioned papers through social media is available at https://www.altmetric.com/explorer/report/c1a1ddfa-6c7d-42b8-8370-41203f1a3f34. Follow us and get engaged!

There are many more changes that have already been made and that are on their way. One significant innovation is the introduction of special issues that will focus on one specific disease. We start this month with an issue on endometriosis. It includes a very nice series of high-class article reviews, expert opinions, and original papers focused on this cutting-edge topic.

We hope you enjoy it and look forward to suggestions for future contributions.

We wish you a good start in 2023!

Stay healthy and safe.

Kind regards,

Prof. Karl Oliver Kagan,

*Editor-in-Chief.*

